# Investigation of Acetone Vapour Sensing Properties of a Ternary Composite of Doped Polyaniline, Reduced Graphene Oxide and Chitosan Using Surface Plasmon Resonance Biosensor

**DOI:** 10.3390/polym12112750

**Published:** 2020-11-20

**Authors:** Fahad Usman, John Ojur Dennis, E M Mkawi, Yas Al-Hadeethi, Fabrice Meriaudeau, Thomas L. Ferrell, Osamah Aldaghri, Abdelmoneim Sulieman

**Affiliations:** 1 Department of Fundamental and Applied Sciences, Universiti Teknologi PETRONAS, Malaysia, Seri Iskandar, Perak 32610, Malaysia; johndennis@utp.edu.my; 2 Department of Physics, Faculty of Science, King Abdulaziz University, Jeddah 21589, Saudi Arabia; emrzog@kau.edu.sa (E.M.M.); yalhadeethi@kau.edu.sa (Y.A.-H.); 3 ImViA EA 7535, Team IFTIM, Université de Bourgogne, 21000 Dijon, France; Fabrice.Meriaudeau@u-bourgogne.fr; 4 Department of Physics and Astronomy, University of Tennessee, 401 Nielsen Physics Building and Joint Institute for Materials Research 1408 Circle Drive Room 219 2641 Osprey Way, Knoxville, TN 37996, USA; tferrell@utk.edu; 5 Physics Department, College of Science, Al-Imam Muhammad Ibn Saud Islamic University, P.O. Box 5701, Riyadh 11432, Saudi Arabia; odaghri@gmail.com; 6 Radiology and Medical Imaging Department, College of Applied Medical Sciences Prince Sattam bin Abdulaziz University, P.O. Box 422, Alkharj 11942, Saudi Arabia; a.sulieman@psau.edu.sa

**Keywords:** surface plasmon resonance sensor, acetone vapour detection, diabetes, doped polyaniline, reduced graphene oxide, chitosan

## Abstract

This work reports the use of a ternary composite that integrates p-Toluene sulfonic acid doped polyaniline (PANI), chitosan, and reduced graphene oxide (RGO) as the active sensing layer of a surface plasmon resonance (SPR) sensor. The SPR sensor is intended for application in the non-invasive monitoring and screening of diabetes through the detection of low concentrations of acetone vapour of less than or equal to 5 ppm, which falls within the range of breath acetone concentration in diabetic patients. The ternary composite film was spin-coated on a 50-nm-thick gold layer at 6000 rpm for 30 s. The structure, morphology and chemical composition of the ternary composite samples were characterized by FTIR, UV-VIS, FESEM, EDX, AFM, XPS, and TGA and the response to acetone vapour at different concentrations in the range of 0.5 ppm to 5 ppm was measured at room temperature using SPR technique. The ternary composite-based SPR sensor showed good sensitivity and linearity towards acetone vapour in the range considered. It was determined that the sensor could detect acetone vapour down to 0.88 ppb with a sensitivity of 0.69 degree/ppm with a linearity correlation coefficient of 0.997 in the average SPR angular shift as a function of the acetone vapour concentration in air. The selectivity, repeatability, reversibility, and stability of the sensor were also studied. The acetone response was 87%, 94%, and 99% higher compared to common interfering volatile organic compounds such as propanol, methanol, and ethanol, respectively. The attained lowest detection limit (*LOD*) of 0.88 ppb confirms the potential for the utilisation of the sensor in the non-invasive monitoring and screening of diabetes.

## 1. Introduction

Diabetes is a disease that occurs due to improper regulation of human blood sugar [1]. Genetics and environmental factors such as population growth, ageing, urbanization, obesity, and physical inactivity are the most common causes of diabetes [2,3]. Some of the complications of diabetes include damage to blood vessels which can lead to heart attack and stroke, and problems with the kidneys, eyes, feet and nerves [4]. The World Health Organization (WHO) has reported around 1.6 million diabetes-related deaths globally in 2016. In addition, the global diabetes prevalence is estimated to rise to 10.2% (578 million) by 2030 and 10.9% (around 700 million) by 2045 as compared to 9.3% (463 million people) in 2019 [4,5,6].

Diabetes is currently diagnosed based on plasma glucose criteria or A1C (glycated haemoglobin) criteria [6]. However, these methods are invasive, painful, and inconvenient [7]. In addition, there is the possibility of contracting diseases and damaging tissues [8]. Also, some of these methods are expensive, require trained personnel, and feature non-real-time detection and laboratory-restricted usage [9]. These necessitate the need for a real-time and non-invasive means of diagnosing diabetes. Exhaled breath acetone has been identified as a good biomarker for the non-invasive diagnosis of diabetes [2]. It was stated that acetone concentration in the human body is generally very low (0.1 ppm–0.8 ppm), while it might be high in the case of metabolism disorders, including diabetes mellitus (DM) (1.8 ppm–5.0 ppm) [10].

The conventional means of acetone vapour detection such as gas chromatography—mass spectrometry (GC-MS) and selected ion flow tube mass spectrometry (SIFT-MS) are capable of detecting traces of acetone vapour with good sensitivity and selectivity. However, these methods are expensive and rely on sophisticated instrumentation, complicated sample collection methods, and are found only at advanced medical institutions [2].

The aforementioned limitations stimulated investigation on biosensor-based detection of acetone vapour [2]. Chemiresistive biosensors are the dominant acetone vapour biosensors due to their cost-effectiveness and many other interesting features [2]. Unfortunately, most of these sensors are based on metal oxide semiconductors (MOS) active sensing layers that operate at high temperatures in addition to cross-sensitivity and lack of stability [11,12]. This increases the power consumption and restricts its feasible utilisation. Furthermore, the response (i.e., resistance) of the sensors is influenced by other ambient factors such as the contact resistance of the electrodes [13].

Generally, one type of biosensor, i.e., optical biosensors, has significant advantages over other types of biosensors due to their higher sensitivity, electrical passiveness, freedom from electromagnetic interference, wide dynamic range, and multiplexing capabilities [14,15,16]. Furthermore, optical biosensors based on surface plasmon resonance (SPR) have additional advantages such as rapid, quantitative, and label-free detection [15]. SPR biosensors have been reported to be functionalised with different active sensitive materials and utilised in the successful detection of some volatile compounds and gases such as benzene, ammonia, chloroform, ethanol, methanol, ethyl benzene, 2-propanol, toluene and acetone vapours [17,18,19]. However, based on our literature search, SPR biosensors have not been optimized for the detection of low concentrations of acetone vapour that could be applied in the non-invasive monitoring and screening of diabetes [19,20].

Recently, a ternary composite comprising of p-toluene sulfonic acid (PTSA) doped polyaniline (PANI), chitosan and reduced graphene oxide (RGO) has been synthesised by our group [21]. The composite was proposed to incorporate the advantageous properties of the individual components [21,22]. Fortunately, the composite has shown improved thermal stability especially below 100 °C, lower optical band gap, higher electrical conductivity, and good dispersibility [21]. More importantly, the ternary composite has demonstrated a better SPR sensing performance compared to the binary composites and the separate components, as shown in Figure S1a–e and Table S1.

This work is aimed at investigating the detection of low concentrations of acetone vapour for possible application in the monitoring and screening of diabetes in exhaled breath using the ternary composite-based SPR sensor. The synergistic effect of the individual components is expected to facilitate the detection of such low concentrations of acetone vapour within the range of 1.8 ppm–5.0 ppm found in diabetic subjects [10].

## 2. Materials and Methods

### 2.1. Chemicals

Ternary composite was synthesised in house; 1-Methyl-2-pyrrolidinone (NMP), p-toluene sulfonic acid or Toluene-4-sulfonic acid monohydrate (PTSA), acetone (99%), ethanol, methanol and propanol were all supplied by Avantis chemicals supply from Merck (Darmstadt, Germany) and Sigma Aldrich (St. Louis, MO, USA). All the chemicals were of analytical grade.

### 2.2. Synthesis of the Ternary Composite

The details of the synthesis procedure can be found in our previous work [21]. In summary, 1 g of chitosan was dissolved in 100 mL of aqueous acetic acid (2% *v*/*v*), stirred for 24 h at room temperature, and kept separately. Another separate homogeneous dispersion of 100 mg RGO in 45 mL of 0.4 M solution of PTSA was prepared by ultra-sonication for 2 h. The two separate components were combined and subjected to continuous magnetic stirring. To the mixture of the separate components, about 50 mL of 0.1 M of aniline (dissolved in 0.4 M PTSA) was added and stirred for 15 min in order to form a homogenous dispersion. Thereafter, 10 mL of 0.15 M APS (dissolved in 0.4 M PTSA) solution was then added to the dispersion drop-wise under constant stirring at a temperature of 0–5 °C. The reaction mixture was kept under constant stirring for an additional 6 h. The greenish-black precipitate obtained was separated and washed until the filtrate became colourless. The final composite was dried at 50 °C for 24 h.

### 2.3. Fabrication of Au/Ternary Composite Sensor Film

Gold (Au) film was deposited on glass cover slips (24 mm × 24 mm × 0.1 mm, Menzel-Glaser, Braunscheig, Germany). The glass slip was cleaned prior to the deposition process. Typically, the slip underwent ultra-sonication in 50% solution of acetone for 5 min followed by rinsing in concentrated acetone and deionised water. This is to remove dirt and any trace of impurities on the surface of the glass slide before any coating process. The glass slide was then coated with a thin film of gold layer using a sputter coater (model SC7640, Quorum Technologies, Newhaven, East Sussex, UK) at 20 mA for 67 s based on previous experience [23]. The gold-coated substrate was then stored in EKB-103 storage box. Subsequently, the thin film of the ternary composites was deposited on top of the gold-coated substrate by preparing 15 mg/mL solution in 1-Methyl-2-pyrrolidinone (NMP). The deposition was conducted using POLOS™ spin coater at 6000 rpm for 30 s. The Au-coated glass substrate with the thin film of the ternary composite deposited on it was then kept in an oven at 40 °C for 1 h to allow the solvent to evaporate.

### 2.4. Characterisation

#### 2.4.1. Spectroscopy

FTIR spectroscopy was carried out on an FTIR spectrometer (Bruker Instruments, model Aquinox 55, Ettlingen, Germany) in the 4000–400 cm^−1^ range using KBr pellets. The optical characterisation has been conducted using UV–Vis spectroscopy (Cary 100 UV-Vis Spectrophotometer from Agilent Technologies, Santa Clara, CA, USA at room temperature on a thin film of the tested materials.

#### 2.4.2. Thermal Analysis

The thermal stability study of the ternary composite was conducted under a nitrogen atmosphere from room temperature up to 850 °C at a heating rate of 10 °C/min.

#### 2.4.3. Morphology and Thickness Measurement

The Field Emission Scanning Electron Microscopy (FESEM) image was obtained at 5 kV using VPFESEM, Zeiss Supra55 VP (Oberkochen, Germany). The thickness measurement was conducted using a surface roughness tester (SV-mutitoyo-3000, Mutitoyo, Aurora, IL, USA) by scratching the film after deposition.

#### 2.4.4. Atomic Force Microscopy (AFM)

The surface of the ternary coated gold film and the surface of the gold film were investigated with atomic force microscopy (AFM, WITec, Ulm, Germany) in order to explore the surface coverage as well as the surface roughness of the sensing layers.

#### 2.4.5. EDX

The energy-dispersive X-ray (EDX or EDS) technique was conducted to determine the constituent elements. This was to allow the accurate description of the possible interaction mechanism between the sensing layer and the analyte.

#### 2.4.6. X-Ray Photoelectron Spectroscopy (XPS)

The functional groups of constituent materials present on the surface of the sensing layers were investigated by X-ray photoelectron spectroscopy (XPS, Thermo logical, K-alpha, Waltham, MA, USA) in order to predict the interaction mechanism between the sensing layer and the acetone vapour. EDX explores elemental composition to the micro-meter range while XPS deals with the near-surface in the order of few nanometres.

### 2.5. SPR Measurement

**Figure 1:**
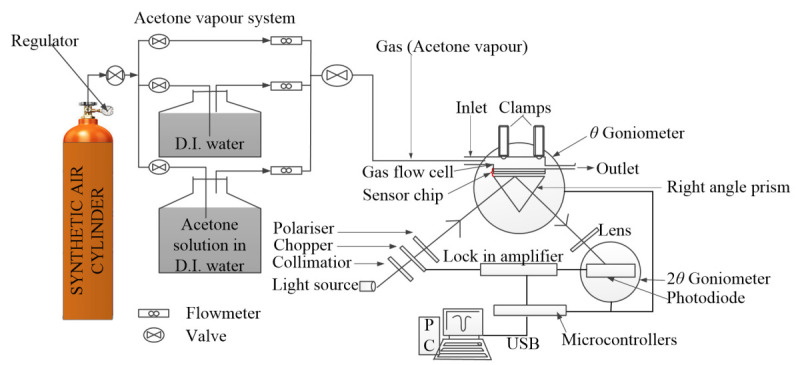
Schematic diagram of the entire SPR response testing setup.

The SPR acetone-sensing properties of the ternary composite were studied using an experimental setup shown as shown in the schematic diagram in Figure 1. A photograph of the actual experimental setup is shown in Figure S2. The setup is based on Kretschmann-Raether configuration. Typically, a 5 mW He-Ne laser source (*λ* = 632.8 nm) was p-polarized using the beam splitter, two polarisers and a pinhole lens, and then directed at an SF11 prism with an Au/ternary-composite coated glass-slide attached at its base. The p-polarized light is reflected through the other facet of the prism after interacting with the gold film. Energy is exchanged between the polarized light waves and surface plasmons generated by the gold film when their wave vectors match at the surface plasmon resonance (SPR) condition. The incident angle of the light at which nearly complete attenuation of the reflected light occurs is called the SPR angle. This is the SPR signal (response) which is detected by a sensitive silicon photodiode. The response was further processed by a lock-in amplifier (SR530, Stanford Research Systems, Sunnyvale, CA, USA) and displayed on a PC. The prism with the Au/ternary film attached was placed on an optical stage driven by a stepper motor (Newport MM3000, ARTISAN TECHNOLOGY GROUP, Champaign, IL, USA) with a resolution of 0.001° in order to allow the p-polarised light to pass through the prism and interact with the gold film for the SPR induction. In order to reuse the prism, both the gold film and the ternary composite film were deposited on a glass cover slip and attached to the base of the prism using a Norland index matching liquid.

An airtight stainless-steel gas test cell (Figure S3) was fabricated in-house with an opening for attaching the glass substrate with the Au/ternary-composite thin film facing into the cell. The test cell has an inlet and outlet ports to pass the gas-phase analytes (acetone vapour and other interfering gases) over the active layer of the glass substrate consisting of the Au/ternary-composite thin film. The analyte gases were introduced into the gas test cell through a plastic tube at optimum volume flow rate. The temperature and relative humidity in the test cell were monitored by a humidity/temperature meter (HT-601C, OEM, Ilioupoli, Greece). All the experiments were conducted at room temperature and the optimum flow rate of the gasses were explored in the range of 50–250 mL/min.

## 3. Results

### 3.1. Morphological Structure, Chemical Composition and the Thermal Stability of the Ternary Composite

**Figure 2:**
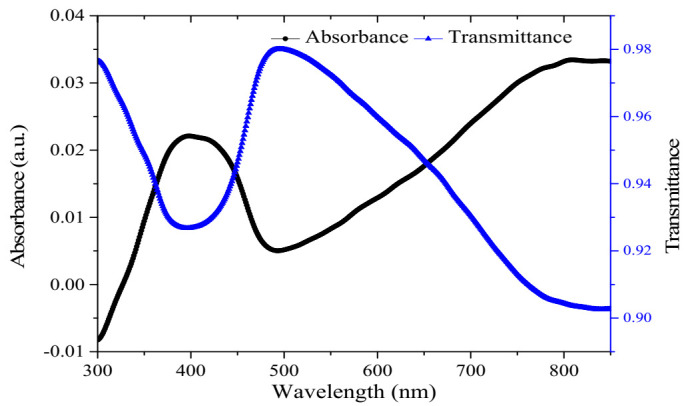
UV-Vis absorption and transmittance spectra vs. the wavelength of the ternary composite film prepared using NMP solvent.

The result for the FTIR and UV-Vis characterisation of the ternary composite has been explained in our previous works [21,24]. Despite the usage of NMP as the dispersion solvent, in this case, the UV-Vis spectra still maintain a similar shape, as shown in Figure 2. However, the absorption spectrum of the ternary composite is more related to the spectrum of CSA doped polyaniline dispersed in meta cresol solvent explained previously [25,26]. The manifestation of the free carrier tail in place of delocalised polarons band and the overlapping of π-π transition in addition to the localised Polaron has been attributed to the greater delocalisation of the polaron band and the elimination of energy gap between ***π*** band and the polaron band, respectively [25,27]. It is worth noting that both the chitosan and the RGO do not present any absorption peaks in the 300 to 850 nm range [28,29]. As such, the observed PANI peaks could be extended to confirm the formation of the ternary composite. In addition, the transparent nature of the film in the visible region indicates the potential for utilizing the ternary composite material for the SPR-sensing application.

In order to confirm the presences of the sensing layer and its composition elements on the surface of the gold-coated glass substrate, FESEM, EDX, and AFM analysis were conducted.

**Figure 3:**
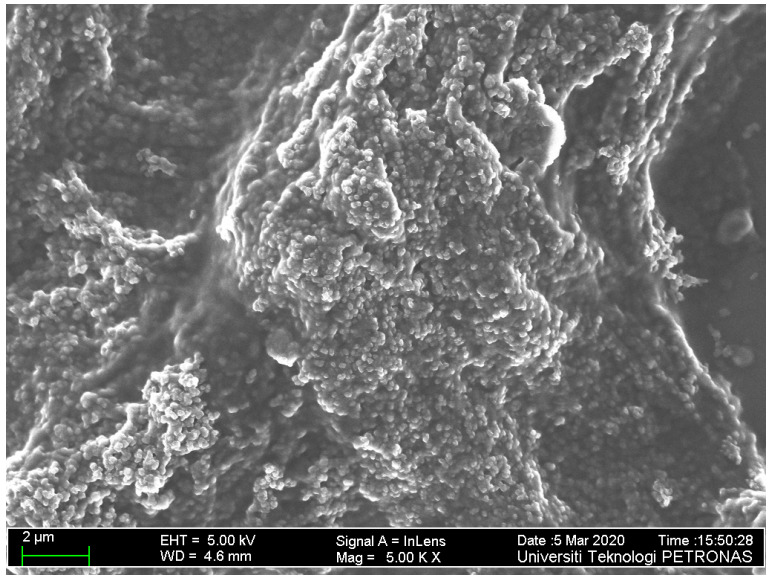
FESEM image showing the morphology of ternary thin film deposited on the gold layer.

FESEM analysis was conducted to observe the surface morphology of the ternary composite layer and is shown in Figure 3. The result for the ternary composite film is in accordance with the result obtained when the material was deposited on a glass substrate in our previous work [21]. Interestingly, clearer features are depicted in this case, which is inconsistent with previous work on PANI-graphene oxide and reduced graphene oxide [30]. This is possibly due to the interaction between the gold layer and the ternary composite layer [31]. In addition, the obvious rough surface could result in better absorption of the analyte of interest on the surface of the sensing layer [32].

Despite this rich information from the FESEM, a clearer picture of this material is further obtained from EDX analysis. EDX identifies the composition of different elements in the samples through an interaction between some source of X-ray excitation and the various samples [33]. EDX could penetrate down to about 2000 nm [34], and as such, Si and Au could also be observed which originate from the substrates and the gold film, respectively. Percentage compositions of the significant peaks are shown in Table 1. The presence of N, C, and O also suggests the presence of the three component materials (PANI, chitosan and RGO) [35,36]. In addition, the observed trace of S originates from the P-toluene sulfonic acid of the PANI and further confirms the presence of PANI [37]. Interestingly, the higher percentage of oxygen content could be attributed to the presence of the abundant OH functional group from chitosan which has the potential to increase the interaction between the analyte and the sensing layer [38].

**Figure 4:**
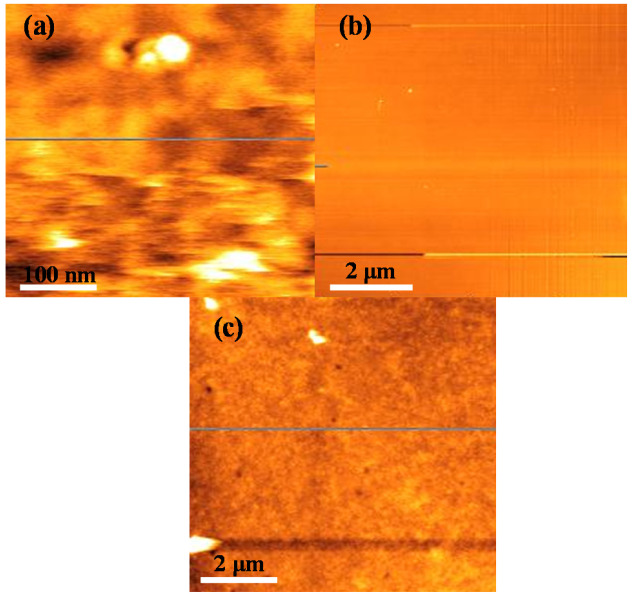
AFM surface morphological images for the (**a**) glass substrate, (**b**) gold thin film, and (**c**) ternary composite thin film, respectively.

The roughness of the sensing layer greatly influences the response of SPR sensors. A smooth surface of the glass substrate and the gold layer is necessary for better surface plasmon excitation and by extension better sensitivity [39], while a reasonable roughness of the sensing layer is required for better adsorption and sensitivity [40]. Figure 4a–c shows the AFM surface morphological images for the glass substrate, gold, and the ternary composite, respectively, and the values for the roughness parameters are presented in Table 2. As shown in the table, the roughness values indicated by Ra and RMS for both the glass substrate and gold layer are sufficiently low for constructing a good SPR sensor [39]. On the other hand, the Ra roughness value for the ternary composite is 5.22 nm, while the RMS value is higher at 10.77 nm. This higher roughness of the ternary composite film could improve the performance of the SPR sensor.

The optimum thickness of the gold film in the Kretschmann configuration of SPR sensors lies within the range of 40–52 nm [15,41]. As shown in Figure S4, the thickness of the gold film deposited on the glass substrate is about 50 nm and, therefore, falls within a suitable range for achieving a highly sensitive SPR sensor.

**Figure 5:**
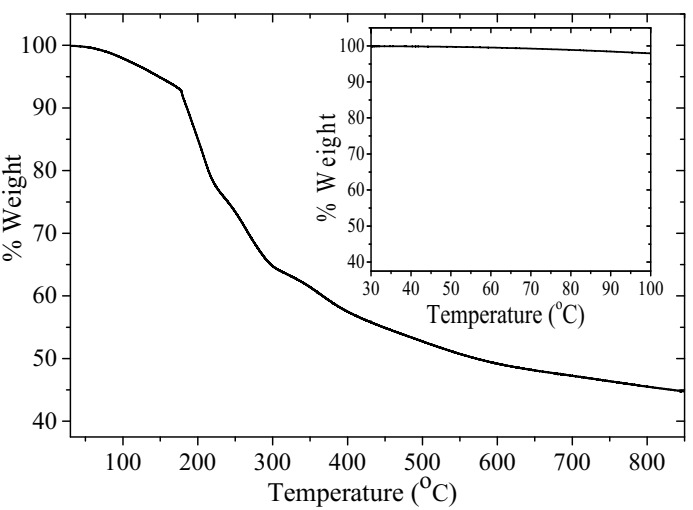
The TGA thermogram of the ternary film in nitrogen obtained at the heating rate of 10 °C/min.

The thermal stability analysis of the ternary composite film is illustrated in Figure 5. It could be observed that the material decomposes with increasing temperature through different decomposition stages. The obvious weight loss up to around 190 °C is attributed to the removal of water molecules and other volatile impurities [42,43]. Moreover, the less intense stages above 190 °C are due to the decomposition of the PANI backbone and other functional groups due to chitosan and RGO [42,43,44]. Fortunately, the weight of the ternary composite film is almost 98% up to about 100 °C (inset of Figure 5) which falls in the interest range of this biosensor. This indicates the applicability of the composite material for realizing the SPR biosensor.

### 3.2. Acetone Sensing Properties of the Ternary Composite

#### 3.2.1. Experimental Response of the SPR Sensor to Different Concentrations of Acetone Vapour in Air 

**Figure 6:**
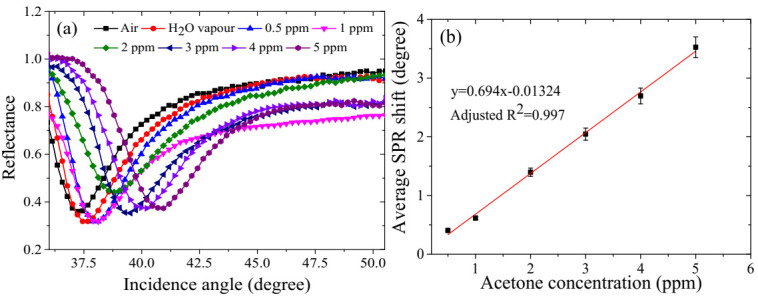
(**a**) Variation of SPR angle due to exposure of the SPR sensor to air, water vapour and various concentrations of acetone vapour from 0.5 ppm to 5 ppm, and (**b**) average SPR angle shift versus acetone concentration (0.5 ppm–5 ppm).

The SPR sensor was exposed to analytes and synthetic air for about 5 min for each run to stabilise the SPR signal and flush out the analytes, respectively [23,45]. In addition, the optimal operating conditions of the SPR sensor were determined to be 29.9 °C temperature, 90% RH, and a flow rate of 150 mL/min. As indicated in Figure 6a and Table S2, the SPR angle has shifted to the greater incidence angle upon exposure to the humidified air (90%RH) as compared to dry air. The same trend was also observed during exposure to different concentrations of acetone vapour in the range of 0.5 ppm to 5 ppm, as shown in the same figure. This could be attributed to the changes in the refractive index of the sensor surface which in turn changed the dielectric constant of the gold film due to the binding of the analyte [15,23]. The repeatability of the SPR sensor was assessed in terms of the standard deviation (S.D.) and the coefficient of variation (COV) of three replicas [23,46,47,48]. As shown in Table S2, both the values of the S.D. and COV are very small, 0.058% and 0.147%; respectively. These confirm the repeatability of the measurement [46,47,49]. The calibration curve of the SPR sensor for acetone detection is shown in Figure 6b. It is observed that the average SPR angle shift is linearly correlated to the acetone vapour concentration in the air with a correlation coefficient of 0.997 and a computed sensitivity value of 0.694 degree/ppm.

#### 3.2.2. Variation of Layer Thickness of the Ternary Composite Films and Lowest Detection Limit (*LOD*)

**Figure 7:**
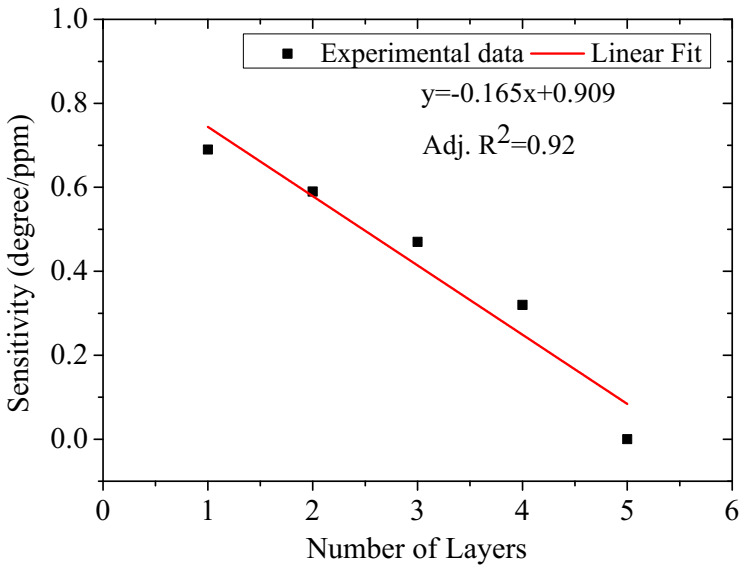
SPR sensor sensitivity versus the number of ternary composite layers.

To investigate the optimum sensitivity of the SPR sensor as a function of layer thickness, the thickness of the ternary composite layer was varied from 1 layer to five layers by repeated spin-coating deposition at 6000 rpm for 30 s for each additional deposited layer. The SPR sensor based on the single-layer demonstrated the best performance, as shown in Figure S5a,b, Table 3, and Figure 7. The single-layer SPR sensor features greater values of sensitivity, full width at half maximum (FWHM), and the figure of merit (FOM), as shown in Table 3. The FOM is evaluated as sensitivity/FWHM. This could be attributed to the decrease in the penetration depth of the surface plasmon wave with an increase in the number of layers [15].

The lowest detection limit (*LOD*) of the ternary based SPR acetone vapour sensor was estimated using Equation (1) [50].

(1) $$LOD=\frac{3\sigma }{S_{n}}$$

where *σ* and *S_n_* stand for the standard deviation of the blank sample and sensitivity, respectively.

The SPR response of the blank sample and the values for the single-layer ternary composite based SPR acetone vapour sensor are shown in Figure S5c and Table S3, respectively. The standard deviation (*σ*) of 10 replicas was evaluated to be about 0.0002. This gives the *LOD* value of about 0.88 parts per billion (ppb).

#### 3.2.3. Selectivity and Detection Mechanism of the Single-Layer Ternary Composite Based SPR Acetone Vapour Sensor

The knowledge of functional groups on the surface of a sensing layer allows an excellent description of the dominant interaction mechanism and the reason for a selective detection of an analyte [51]. XPS analysis is usually employed in exploring the constituent elements on the surface of a material. The presence of some important functional groups such as OH, NH, C=O, and sulfonate groups has been confirmed on the surface of the ternary composite presented in our previous work [21]. These functional groups can play a vital role in the selective detection of acetone. Interestingly, a similar result is also observed on the surface of the ternary composite material deposited on top of the gold film. The graphs and the assignments of functional groups are presented in Figure S6 and Table S4, respectively.

**Figure 8:**
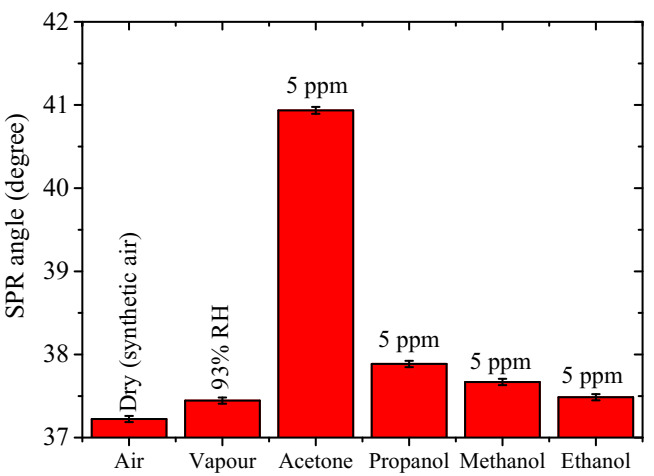
Selectivity of the ternary sensing layer to 5 ppm acetone vapour as compared to dry air, humid air (89%RH), and 5 ppm concentrations of propanol, methanol, and ethanol vapours.

The selectivity of the single layer ternary composite-based SPR sensor to acetone vapour was confirmed by investigating and comparing its response in dry air, water vapour, and to the same concentration of 5 ppm each of the propanol, methanol, and ethanol vapours. The selectivity graph is shown in Figure 8, where it is observed that the maximum SPR angle attained in air and water vapour is about 93% RH, and 5 ppm of acetone, propanol, methanol and ethanol vapours are 37.22°, 37.45°, 40.94°, 37.89°, 37.67° and 37.49°, respectively. The response to the 5-ppm acetone vapour is about 87%, 94%, and 99% higher compared to the same concentration of propanol, methanol, and ethanol, respectively. Furthermore, the insensitivity of the sensing layer to humidity indicates the potential applicability of the ternary composite-based SPR sensor for monitoring or screening for diabetes through the direct exposure of the sensor to exhaled human breath to detect acetone concentrations.

#### 3.2.4. Comparison between the Ternary Composite Based SPR Biosensor and other Published Optical Based Acetone Vapour Biosensors

Based on our review, the majority of the acetone vapour biosensors investigated for the non-invasive monitoring and screening of diabetes were based on the metal oxide semiconductor (MOS) [2], which is in accordance with a recent review [52]. Based on the limitations of the MOS sensors and the advantages of the optical sensors mentioned in the introduction, this work is only compared with other optical acetone sensors and summarised in Table 4.

**Table 4:** Summary of published works on optical acetone vapour sensing and comparison with the proposed ternary composite of PANI, chitosan, and the RGO-based SPR biosensor.

Sensing Layer	Explored Range (ppm)	Mechanism	*LOD* (ppm)	Operation Temperature	Reference
Nano-La_2_O_3_	0.19–140	Cataluminiscence	0.08	361 °C	[61]
Polydimethylsiloxane (PDMS) filled negative axicon	0–200	Fibre optics	2.19	RT	[60]
Tin- Doped Gallium Oxide	100–100,000	Photoluminiscence	N/A	RT	[59]
γ-CuBr nanocrystals	50–500	Fibre optics	N/A	RT	[58]
Nicotinamide adenine dinucleotide (NADH)	0.02–5.3	Fluorescence	N/A	N/A	[62]
Reduced Graphene Oxide/Maghemite	100,000–500,000	SPR	N/A	RT	[19]
Leaning pillar substrates	8000–250,000	Raman shift	3299	N/A	[57]
Porapak QS	N/A	CRDS	0.159	RT	[63]
Catalyst	0.4–17.88	Plasma-assisted cataluminescence	0.05	120 °C	[64]
Resorcinol on Nafion	N/A	Optode (absorption)	<1	60 °C	[56]
PDMS	N/A	Micro-ring resonator	200	N/A	[55]
PDMS	N/A	Micro-ring resonator	200	RT	[54]
Poly(2-vinyl pyridine)	0.1–5 20–100	Micro-ring resonator	0.017	RT	[53]
Ternary composite of PANI, chitosan and RGO	0.5–5	SPR	0.00088	RT	This work

N/A = Not available. RT = Room temperature.

The optical-based biosensors for the detection of acetone gas feature one or more problems as presented in Table 4. These problems range from high *LOD* (>1.8 ppm, diabetes threshold), high operation temperature and inappropriately explored ranges to poor selectivity. In addition, most of the biosensors were not optimised for the detection of acetone vapour for diabetes interest [19,53,54,55,56,57,58,59,60,61]. As such, many important factors such as humidity effects were left unexplored. Moreover, some of the sensors are more promising in other applications, e.g., the NADH-based sensor in lung cancer sensing [62]. Furthermore, the sample cavity enhanced spectroscopy (CRDS)-based sensors are hindered by their time consumption due to the pre-concentration requirement [63]. In the present work, better properties are observed for the ternary-based SPR biosensor. More importantly, the biosensor can achieve a low detection limit down to 0.88 ppb, which is far below the diabetes threshold, in addition to its excellent selectivity. This indicates its potential applicability in the detection of exhaled breath acetone for non-invasive monitoring and screening of diabetes.

## 4. Conclusions

In this work, a highly sensitive SPR sensor based on a ternary composite comprised of PANI, chitosan, and RGO was successfully developed. The aim was to investigate the potential applicability of the sensor for the detection of acetone vapour at low concentrations from 1.8 ppm to 5 ppm for the non-invasive monitoring and screening of diabetes. Analytical techniques such as FTIR, UV-VIS, FESEM, EDX, AFM, XPS, and TGA were employed to characterise the ternary composite-sensing layer. Excellent thermal stability of the ternary layer has been observed up to 100 °C, which is above the operational temperature of the SPR sensor. Also, the results revealed that the SPR sensor could detect the acetone vapour down to 0.88 ppb, which is far below the threshold for diabetes, with a sensitivity of about 0.69 degree/ppm and a correlation coefficient of 0.997. The single-layer ternary composite SPR sensor showed superior selectivity, repeatability, reversibility, and stability with a response to acetone which is 87%, 94%, and 99% higher compared to common interfering volatile organic compounds such as propanol, methanol, and ethanol, respectively. Collectively, these results have indicated the possibility of realising a non-invasive ternary composite of a PANI, chitosan, and RGO-based SPR biosensor for monitoring and screening for diabetes via direct exposure of the sensor to human exhaled breath to measure the concentration of acetone.

## Figures and Tables

**Table 1:** The elemental composition of the ternary composite film from EDX analysis.

Element	Weight (%)	Atomic (%)
C	43.61	50.49
N	4.78	4.75
O	51.32	44.62
S	0.29	0.13

**Table 2:** The values for the roughness parameters for glass, gold, and ternary film surfaces.

Material	Ra (nm)	RMS (nm)
Glass	0.16	0.22
Gold	2.13	4.05
Ternary	5.22	10.77

**Table 3:** Performance characteristics of ternary SPR acetone vapour sensor based on 1, 2, 3, 4, and 5 layers of the ternary composite film.

Number of Layers	FWHM (degree)	Sensitivity (degree/ppm)	FOM (per ppm)
1	2.96	0.69	0.23
2	6.26	0.59	0.09
2	8.57	0.47	0.05
4	11.63	0.32	0.03
5	indefinite	0	0

## Supplementary Materials

The following are available online at https://www.mdpi.com/2073-4360/12/11/2750/s1, Figure S1: Comparison of simulated SPR curves between the ternary composites, binary composites and separate components, Table S1: SPR-sensing performance of the ternary composites, binary composites and separate components, equations of surface plasmon wave penetration depth (Equations (S1) and (S2)), Figure S2: Picture of the experimental SPR setup, Figure S3: Gas measuring cell, Table S2: Data for the ternary based SPR sensor, Figure S4: Thickness of gold thin film deposited at 20 mA, 67s estimated using a surface roughness tester, Figure S5: (a) SPR curves of different layers of the ternary based SPR sensor; (b) SPR angle shift versus the acetone concentration; (c) blank SPR response, Table S3: Blank sample response, Figure S6: XPS spectra for the C1s, O1s, N1s and S2p peaks of the ternary thin layer, Table S4: Assignment of the C1s, O1s, N1s and S2p peaks for the ternary thin layer.
