# Supplementary material for: Investigation of Acetone Vapour Sensing Properties of a Ternary Composite of Doped Polyaniline, Reduced Graphene Oxide and Chitosan Using Surface Plasmon Resonance Biosensor
Source: Polymers (Basel). 2020 Nov 20;12(11):2750. doi: 10.3390/polym12112750 (PMC7699882; doi:10.3390/polym12112750)
Supplement: Supplementary file 1 [file polymers-12-02750-s001.pdf]

Investigation of acetone vapour sensing properties of a ternary composite of doped polyniline, reduced graphene oxide and chitosan using surface plasmon resonance biosensor (Supporting document)

S1. Introduction:

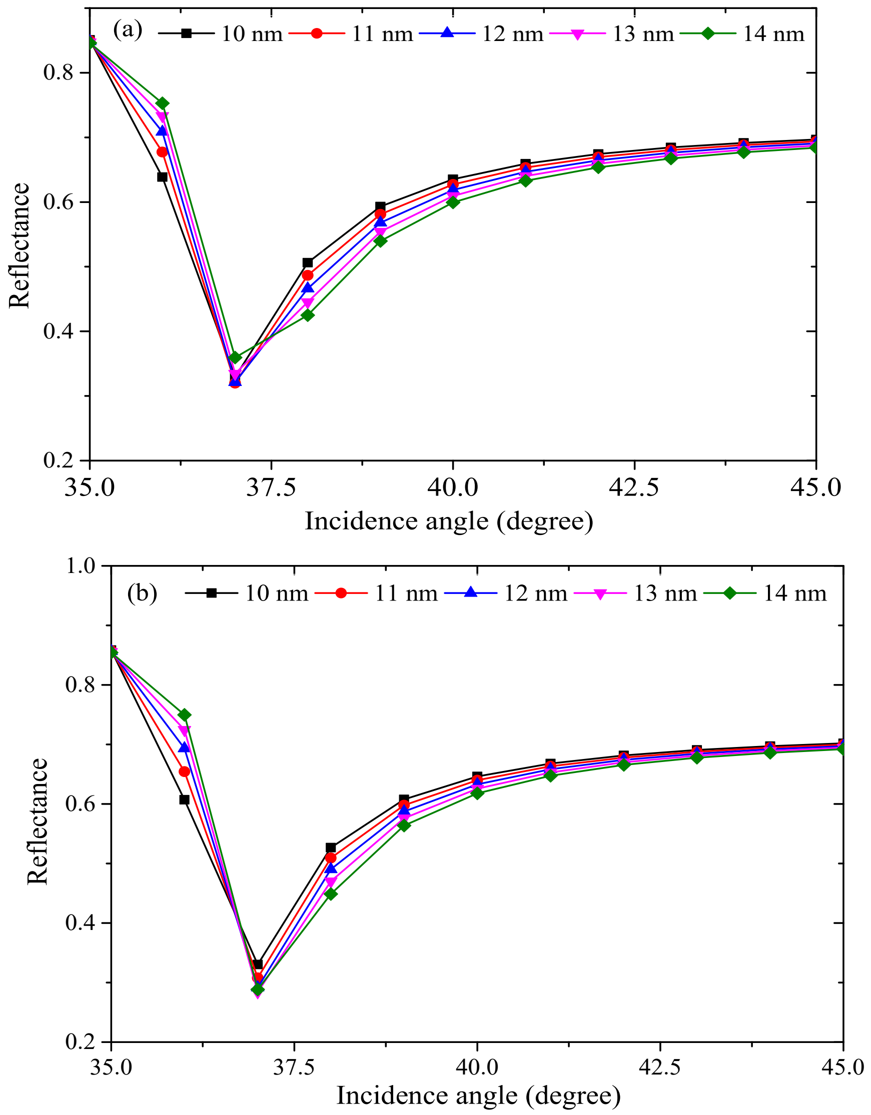

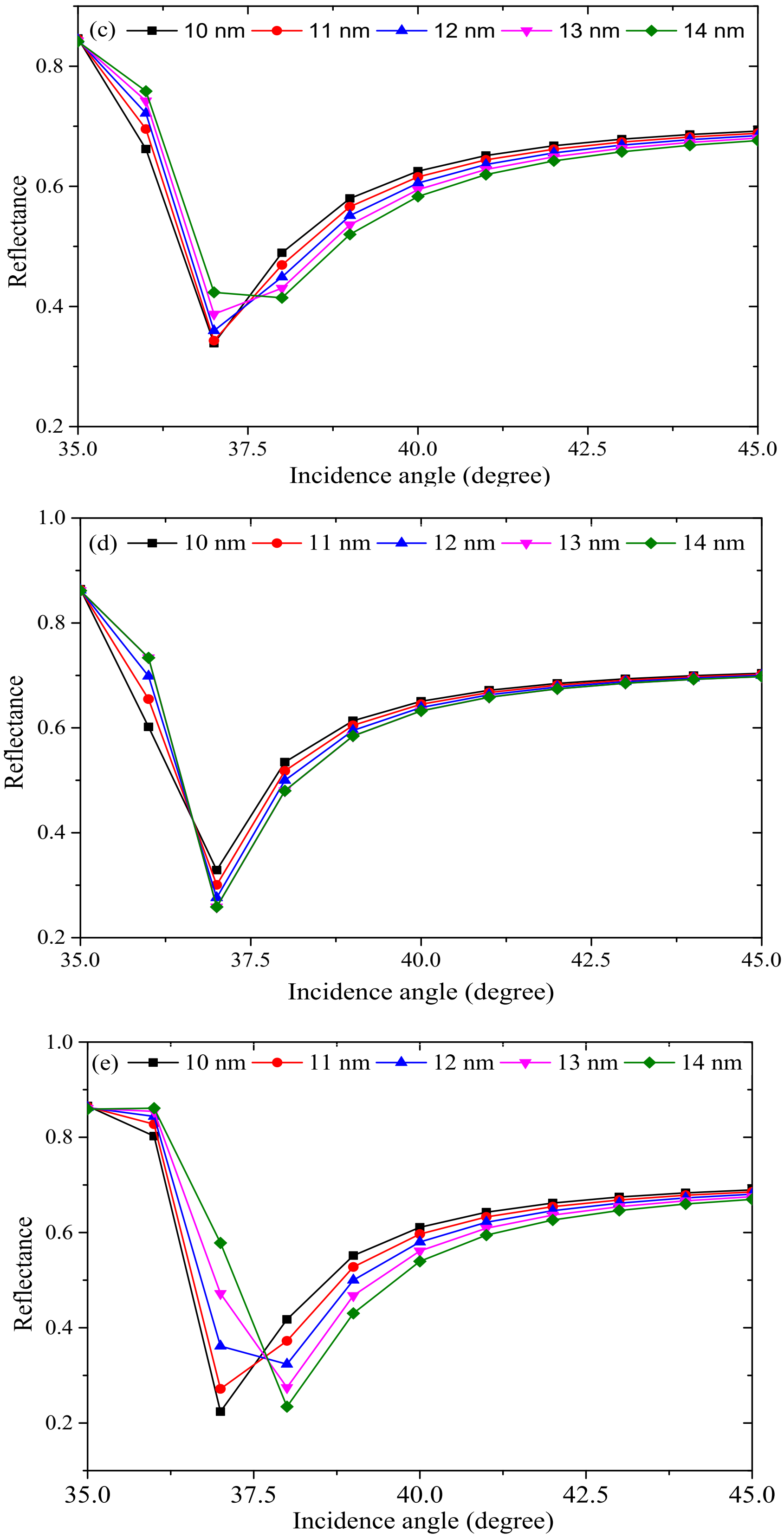

Figure S1. The simulated SPR curves for the (a) PANI, (b) PANI-chitosan, (c) PANI-RGO, (d) Ternary and the (e) Chitosan-PEG composites generated at 10 nm, 11 nm, 12 nm, 13 nm and 14 nm thicknesses using the MATLAB SPR simulation program by ITMA

Table S1. Performance of the simulated SPR biosensors based on PANI, PANI-chitosan, PANI-RGO, Ternary and the Chitosan-PEG composites

Material	δ_d (nm)	δ_m (nm)	FWHM (degree)	SNR (per degree)	Experimental sensitivity (degree/ppm)
PANI	180.08	28.58	2.61	0.38	-
PANI-chitosan	194.72	28.42	2.38	0.42	-
PANI-RGO	168.17	28.73	3.01	0.33	-
Ternary	199.55	28.37	2.14	0.47	0.69
Chitosan-PEG	181.83	28.56	2.44	0.41	0.35

Penetration Depth of Surface Plasmon Waves (δ)

It is defined as the distance from the interface of metal-dielectric at which the amplitude of the field becomes 1/*e* of the value at the interface [38]. The penetration depth in the dielectric gives us a measure of the length over which surface plasmon is sensitive to the changes in the refractive index of the dielectric medium, while the penetration depth into metal gives us an idea of the thickness of the metal film required for the coupling of light incident from the other interface of the metal film [38]. Equation S1 and S2 represents the penetration depth through the gold (δ_m) and the materials (δ_d) adjacent to the gold film, respectively [57].

$$\delta_m = \frac{\lambda_0}{2\pi}\left[\frac{\varepsilon_m^{'} + \varepsilon_d}{(\varepsilon_m^{'})^2}\right]^{1/2} \tag{S1}$$

$$\delta_d = \frac{\lambda_0}{2\pi}\left[\frac{\varepsilon_m^{'} + \varepsilon_d}{(\varepsilon_d)}\right]^{1/2} \tag{S2}$$

Where λ_0 is the free space wavelength ε_m' and ε_d are the real part dielectric constant of the plasmonic material (gold) and the material adjacent to the gold, respectively.

S2. Materials and Methods:

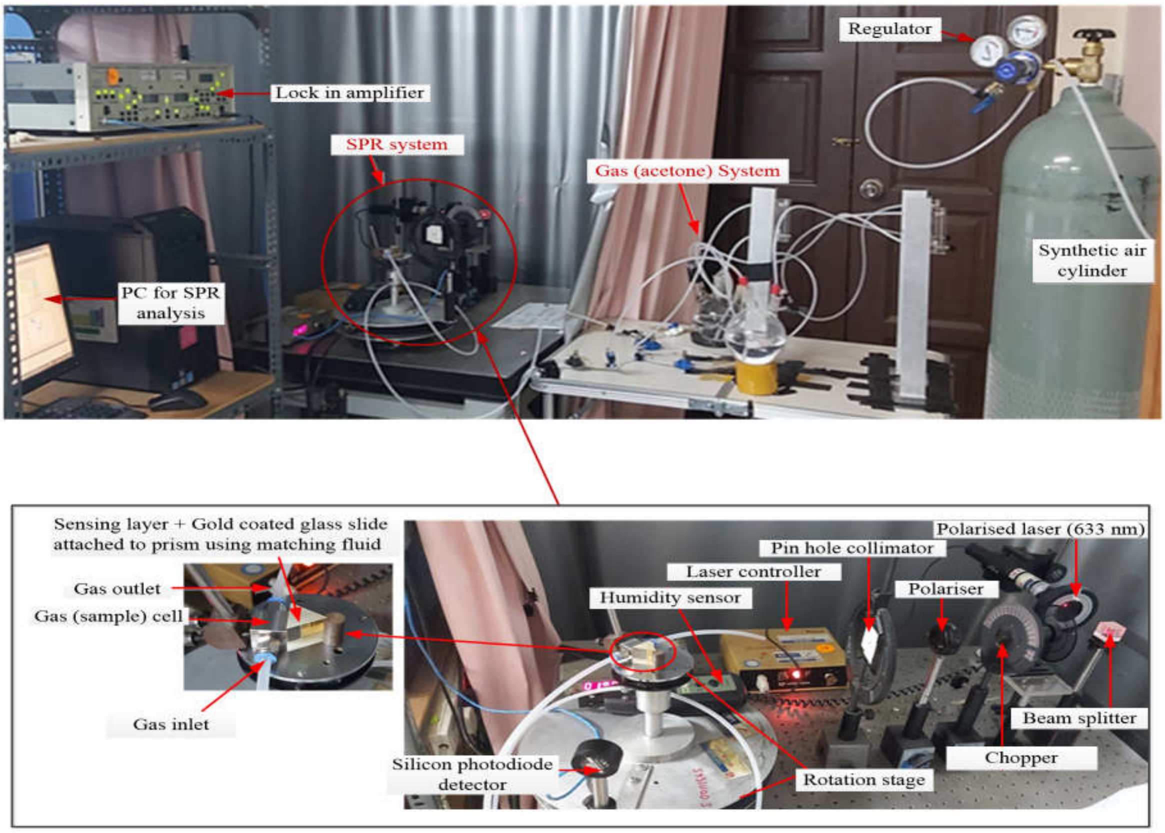

Figure S2. Picture of the experimental SPR setup

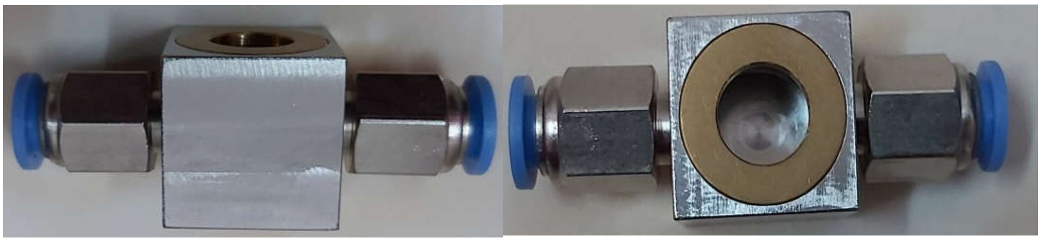

Figure S3. Stainless steel gas measuring cell

S3. Results and Discussion:

Table S2. Results for the measurement of ternary based SPR angle shift due to air, water vapour and the various concentrations of the acetone vapour (0.5-5 ppm).

Concentration of acetone vapour (ppm)	SPR angle, θ (degree) 1st Run	SPR angle, θ (degree) 2nd Run	SPR angle, θ (degree) 3rd Run	Average θ	Average σ	Coefficient of variation (σ/Average)	SPR shift due to acetone, Δθ (degree)
Air	37.2231	37.2231	37.22<br>44	37.2235	0.00061	0.00002	
H_2O vapour	37.4455	37.4455	37.44<br>50	37.4453	0.00024	0.00001	0
0.5	37.8929	37.8700	37.78<br>40	37.8490	0.04688	0.00124	0.4037
1	38.1196	38.0721	37.99<br>24	38.0614	0.05248	0.00138	0.6161

2	38.8063	38.9446	38.7750	38.8420	0.07369	0.00190	1.3967
3	39.5058	39.3473	39.6187	39.4906	0.11132	0.00282	2.0453
4	40.2167	40.2156	39.9906	40.1410	0.10633	0.00265	2.6957
5	40.9357	40.9052	41.0719	40.9709	0.07247	0.00177	3.5256
				38.7529625	0.0580025	COV=0.00147	

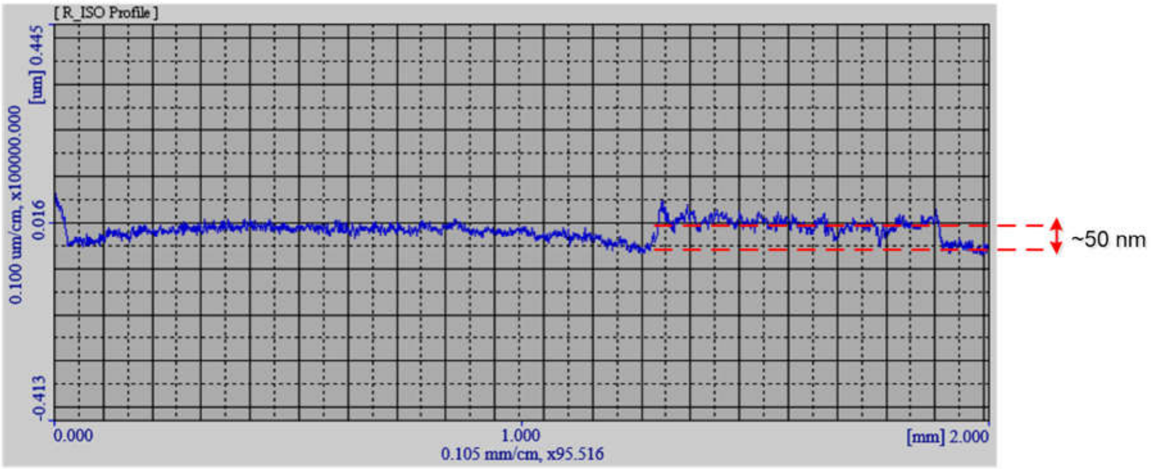

Figure S4. Result for the thickness measurement of a gold thin film deposited at 20 mA, 67s using a surface roughness tester

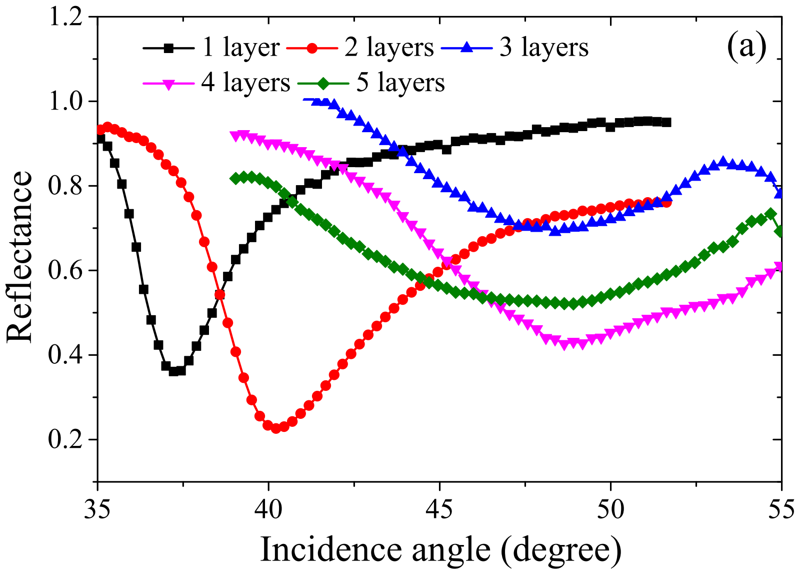

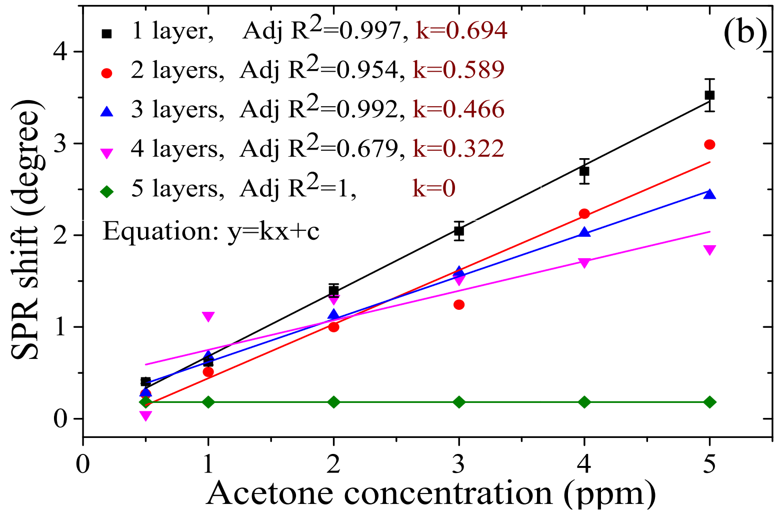

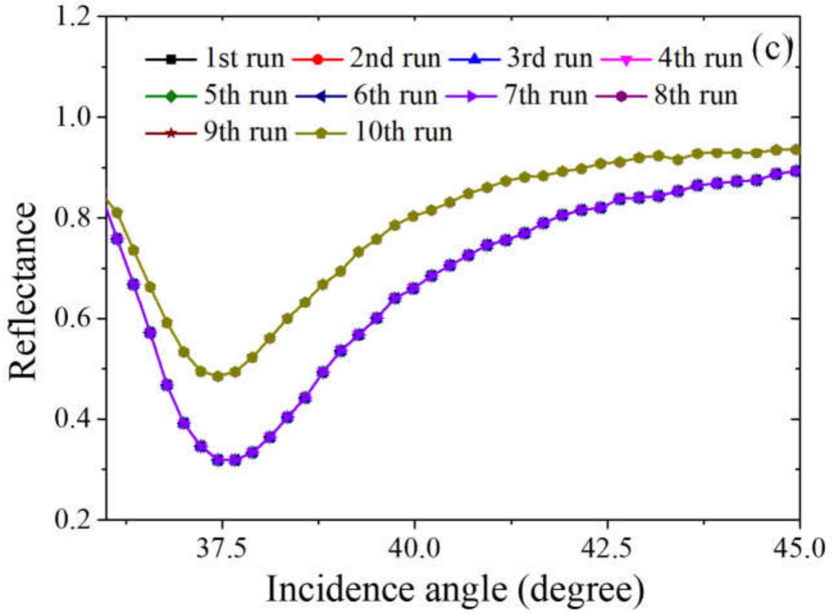

Figure S5 (a) SPR curves of different layers ternary based SPR sensor in synthetic air (1-5 layers), (b) SPR angle shift versus the acetone concentration (0.5-5 ppm) for 1,2,3,4 and 5 layers of ternary based SPR sensor and (c) ternary based blank SPR response for the estimation of limit of detection (LOD).

Table S3. Blank sample response to 1 layer ternary SPR acetone vapour sensor

Number of runs	SPR angle (degree)
1	37.4455
2	37.4455
3	37.4455
4	37.4455
5	37.4455
6	37.4455
7	37.4455
8	37.4455
9	37.4460
10	37.4450

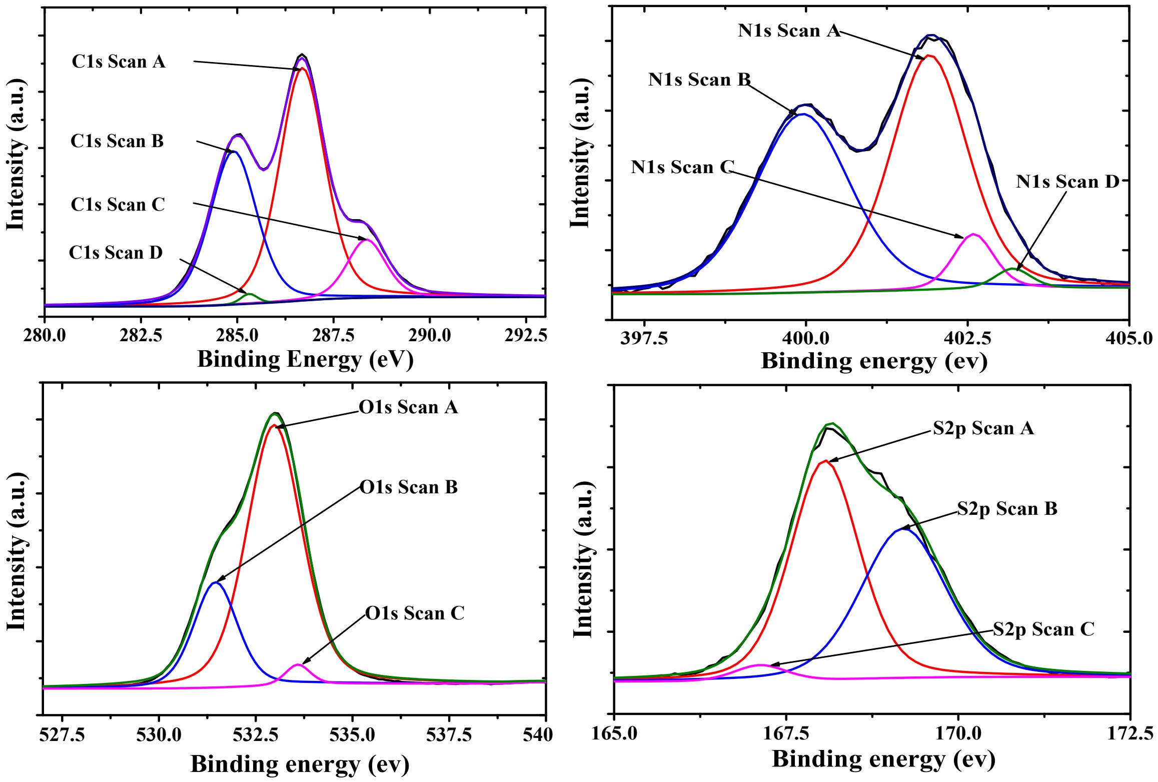

Figure S6. XPS spectra of the C1s, O1s, N1s and S2p peaks for the single layer ternary composite thin film

Table S4. Assignment of the C1s, O1s, N1s and S2p peaks for the single layer ternary composite thin film

Name	Peak(eV)	Assignment
C1s A	286.69	C-O
C1s B	284.90	Contamination, C-C or C-H
C1s C	288.35	C=O
C1s D	285.31	C-NH, C- NH2 or C=C
N1s A	401.91	Oxidized amine
N1s B	399.95	Benzenoid di-amine Nitrogen (-NH-)
N1s C,	402.59	Protonated Imine (-N+)
N1s D	403.20	Protonated Imine (-N+)
O1s A	532.98	C=O
O1s B	531.46	C-OH
O1s C	533.59	C=O
S2p C	167.12	Sulfonate group
S2p A	168.06	Neutral sulfonic acid substituent
S2p B	169.20	Neutral sulfonic acid substituent
